# Tramadol and postoperative sore throat after tracheal intubation in thyroid surgery - a randomized controlled trial

**DOI:** 10.1186/s12871-025-03487-z

**Published:** 2025-12-22

**Authors:** Zegeng Su, Junting Huang, Xiaoling Xu, Bo Yang, Xiuyun Lv, Shuncai Zhang, Xiaohan Wang, Erbin Chen, Yun Xu, Yiting Lin, Xiaoyan Huang, Yongshun Su, Lingui Li, Yuhua Yin, Zheyuan Guo, Litian Tong, Manli Huang, Xiang Xu

**Affiliations:** 1 Department of Anesthesiology, Cancer Hospital of Shantou University Medical College, 7 Raoping Road, Shantou, Guangdong, 515000 China; 2 Department of Thoracic Surgery, Cancer Hospital of Shantou University Medical College, 7 Raoping Road, Shantou, Guangdong, 515000 China

**Keywords:** Postoperative sore throat, Tramadol, Thyroid surgery, Tracheal

## Abstract

**Background:**

Tramadol is widely employed as part of multimodal perioperative analgesia protocols. Despite its extensive clinical use, no existing literature has specifically investigated the effect of intravenous tramadol on postoperative sore throat (POST) following endotracheal intubation. To address this knowledge gap, we conducted a clinical study evaluating the efficacy of intravenous tramadol in reducing the incidence of POST among patients undergoing tracheal intubation for thyroid surgery.

**Methods:**

We randomized 171 American Society of Anesthesiologists (ASA) physical status I-II female patients scheduled for thyroidectomy into three groups: Control: Standard care without tramadol; Pre-tramadol: Received intravenous tramadol (1 mg/kg) 15 min preoperatively; Post-tramadol: Received intravenous tramadol (1 mg/kg) at the initiation of surgical wound irrigation. The primary outcome was the incidence of POST at 24 h after extubation. Secondary outcomes included the incidence of POST in the post-anesthesia care unit (PACU) and at 4 h after extubation.

**Results:**

The incidence of POST at rest was 7.1% for Control group, 7.0% for Pre-tramadol group and 1.8% for Post-tramadol group at rest (*p* = 0.342) at 24 h after extubation; The incidence of POST on movement were 48.2%, 63.2% and 49.1% (*p* = 0.201) at 24 h after extubation, respectively. Similar results also appeared at PACU and 4 h after extubation.

**Conclusions:**

Intravenous tramadol (1 mg/kg) did not significantly reduce the incidence of POST following tracheal intubation in patients undergoing scheduled thyroidectomy.

**Trial registration:**

Date of registration: 05/08/2021. ClinicalTrials.gov; Identifer: NCT04991493. URL: https://clinicaltrials.gov/study/NCT04991493.

## Introduction

POST occurs in 19.4%−71.8% of intubated patients [1–3], making it both common and clinically relevant as patients' sixth-ranked postoperative concern [4]. POST risk factors encompass patient characteristics (female gender [5]), surgical factors (thyroid surgery [3, 6]) and technical variables (larger tube size [6], double-lumen tubes [7], high cuff pressures [8] or coughing during extubation [5]).

To mitigate POST, multiple pharmacological and non-pharmacological interventions have demonstrated efficacy [8, 9]. Pharmacological approaches include intravenous lidocaine, dexamethasone and non-steroidal anti-inflammatory drugs (NSAIDs), all of which reduce the incidence of POST have each been shown to decrease the incidence of POST. Effective non-pharmacological strategies encompass smaller endotracheal tubes, tube lubrication and video laryngoscopy.

Tramadol provides postoperative analgesia through four complementary mechanisms: μ-opioid agonism (primary effect), monoamine (noradrenaline/serotonin) reuptake inhibition, N-Methyl-D-Aspartic Acid (NMDA) receptor antagonism and local anesthetic activity. This multimodal pharmacology yields effective pain control with potentially fewer classic opioid side effects [10].

Clinical studies demonstrate tramadol's topical effectiveness across populations: it reduced POST in pediatric tonsillectomies [11] and, as a preoperative gargle, lowered both incidence and severity of sore throat for 24 h postoperatively in adults [12]. This evidence underscores its role in procedure-specific POST prophylaxis.

Despite intravenous tramadol's established analgesic use, its effectiveness in preventing POST after thyroidectomy remains unproven. This study assesses its impact on the incidence of POST in thyroid surgery patients.

## Materials and methods

### Study design

This single-center, randomized, controlled, double-blinded trial adhered to the principles outlined in the Declaration of Helsinki and obtained approval from The Cancer Hospital, Shantou University Medical College Human Ethics Committee (Shantou, China, No.2021059, 20,210,701).

All participants involved provided written informed consent prior to their enrollment. This randomized controlled trial was registered in ClinicalTrials.gov (ID: NCT04991493, URL: https://clinicaltrials.gov/study/NCT04991493.). The study followed the Consolidated Standards of Reporting Trials (CONSORT) reporting guideline.

### Study population

Female patients with American Society of Anesthesiologists physical status I or II, aged 18–65 years and undergoing thyroidectomy(non-endoscopic) with endotracheal intubation were included in the trial. Patients with history of thyroid surgery, history of preoperative sore throat; pregnancy; retrosternal goiter; thyroid tumor invading trachea need trachea reconstruction; potentially difficult airway; clinical diagnosis of chronic sore throat; clinical diagnosis of psychosis; potential tracheotomy, transfer to intensive care unit after operation, contraindications to tramadol, body mass index (BMI) ≥ 30 kg/m^2^ were excluded. The dropout criteria included withdrawal of consent and other serious perioperative adverse events.

### Intervention

The grouping and medication were as follows: Control group, 5 ml normal saline (in the 5 ml syringe labeled as A) was injected intravenously 15 min before induction of anesthesia and 5 ml normal saline (in the 5 ml syringe labeled as B) was injected intravenously at the initiation of surgical wound irrigation; Pre-tramadol group, 5 ml of tramadol (Tramadol Hydrochloride Injection, Grunenthal GmbH, in the 5 ml syringe labeled as A) containing 1 mg/kg was injected intravenously 15 min before induction of anesthesia and 5 ml normal saline (in the 5 ml syringe labeled as B) was injected intravenously at the initiation of surgical wound irrigation; Post-tramadol group, 5 ml normal saline (in the 5 ml syringe labeled as A) was injected intravenously 15 min before induction of anesthesia and 5 ml of tramadol containing 1 mg/kg (in the 5 ml syringe labeled as B) was injected intravenously at the initiation of surgical wound irrigation.

### Randomization and Masking

Enrolled patients were allocated in a 1:1:1 randomization ratio to Control group, Pre-tramadol group or Post-tramadol group using a block randomization by using R-language (version 4.1.1, package: blockrand, version:1.5). The allocation sequence was created by an off-site statistician who was not involved in study enrolment and allocation concealment was maintained using opaque envelope. Two nurses will open opaque envelopes to verify and prepare injection solutions (two 5 ml syringe label as A and B).

### Procedure

The study was carried out by three investigators in a blinded manner as follows: The first investigator prepared each test solution in a syringe and was also responsible for group allotment. The second investigator (Responsible anesthesiologist), who was blinded to performed the intravenous injection, performed anesthesia and recorded the variables during anesthesia. The third investigator, who was also blinded to recorded the postoperative variables.

After the participants enter the operating room to confirm their information, two nurses will open opaque envelopes to verify and prepare injection solutions. After the administration of 100% oxygen at 5 L/min for several minutes, anesthesia was induced with propofol (2 mg/kg), fentanyl(3 μg/kg), midazolam(0.05 mg/kg) and atracurium (0.5 mg/kg). Video laryngoscope performed 4 min after atracurium injection. Endotracheal intubation was performed using endotracheal tube with 6.5 mm tracheal tube lubricated with physiological saline. The high-volume, low-pressure cuff was inflated until no air leak could be heard with peak airway pressure at 20 cm H_2_O. Inhalational anesthetics, lidocaine and dexamethasone were systematically excluded from all anesthetic protocols.

General anesthesia was maintained with propofol and remifentanil. atracurium administered as required. Controlled mechanical ventilation (Fabius Tiro, Dräger,Lübeck, Germany) with an initial tidal volume of 8 mL/kg and respiratory frequency of 10 breaths/min was adjusted to maintain end expiratory carbon dioxide between 35–45 mmHg. Meanwhile, all patients were intravenously given tropisetron hydrochloride injection 5 mg for the prevention of nausea and vomiting and fentanyl (1 μg/kg) for prevention of incision pain at the initiation of surgical wound irrigation.

When the patients were fully awake, the endotracheal tube cuff was fully deflated and the endotracheal tube was removed after gentle suctioning of oral secretions. All patients received oxygen via a face mask after surgery.

POST and other variables were evaluated in the operating room, 4 and 24 h after extubation. At the time of the first evaluation, patients with a Ramsay Sedation Score 8 of 2 (cooperative, oriented and tranquil) or 3 (responding to commands only) were evaluated.

### Assessment instruments

POST: Defined as persistent pain localized to the pharyngeal or laryngeal region on movement (swallowing movement) or at rest, using a four-point scale (0–3): 0, no sore throat; 1, mild sore throat (less severe than with a cold); 2, moderate sore throat (similar to that noted with a cold) and 3, severe sore throat (more severe than with a cold) [5, 13, 14]. Discomfort of throat: A broader subjective sensation including dryness, itching, or foreign body sensation on movement (swallowing movement) or at rest, using a four-point scale: 0, no discomfort, 1, mild discomfort, 2, moderate discomfort and 3, severe discomfort [14]. The severity of hoarseness, the grading was as follows: 0, no hoarseness; 1, slight hoarseness; 2, severe hoarseness; 3, cannot speak because of hoarseness [5]. Visual analogue scale (VAS) scores for incision pain (linear 10 cm, starting from 0 = no pain to 10 = worst pain imaginable) were recorded at rest and on movement (swallowing movement). Additional analgesics were not administered until completion of the first evaluation.

### Outcomes

The primary outcome was the incidence of POST at rest and on movement (swallowing movement) at 24 h after extubation. The secondary outcomes encompassed several factors, such as the incidence of POST in PACU after extubation, as well as at 4 h after extubation, the severity of hoarseness, the intensity of POST, the incidence of nausea and vomiting and additional analgesics requirement.

### Sample size calculation

Previous studies have shown that the incidence of POST at 24 h after thyroid surgery is 58% in women [3]. The sample size was based upon previously published study [2]; assuming α = 0.05, a sample size of 153 was needed to give greater than 80% power to detect a decrease in the incidence of POST from 58% in the Control group to 34.8% in the Pre-tramadol group and Post-tramadol group. To compensate for potential dropouts, we enrolled 57 patients in each group.

### Statistical analysis

Demographic data, duration of the operation, the time needed for laryngoscopy (time from opening the mouth to placement of the endotracheal tube), number of tracheal intubations, Mallampati grading, grades of laryngoscopic view, intraoperative head movements, coughing or bucking in intraoperative period, coughing or bucking before extubation, coughing or bucking due to deflating cuff during extubation, coughing or bucking on the endotracheal tube, blood staining on the endotracheal tube after extubation, cough after extubation, trachea compressed by thyroid gland, left thyroidectomy, right thyroidectomy and thyroid isthmus resection as well as lymph node dissection were recorded by responsible anesthesiologist.

Continuous variables are presented as mean (SD) or median (IQR), depending on whether the data were distributed normally or not. Categorical variables expressed as numbers (percentages). The normal distribution of data was evaluated using the Shapiro–Wilk test and the Levene method.

was used to test the homogeneity of variance. Chi-square test or Fisher’s exact test was used as appropriate for categorical variables and one-way analysis of variance or Kruskal–Wallis test for continuous variables.

All statistical analyses were performed with use of R-language (version 4.1.1).

## Results

### Medical and demographic characteristics

**Fig. 1:**
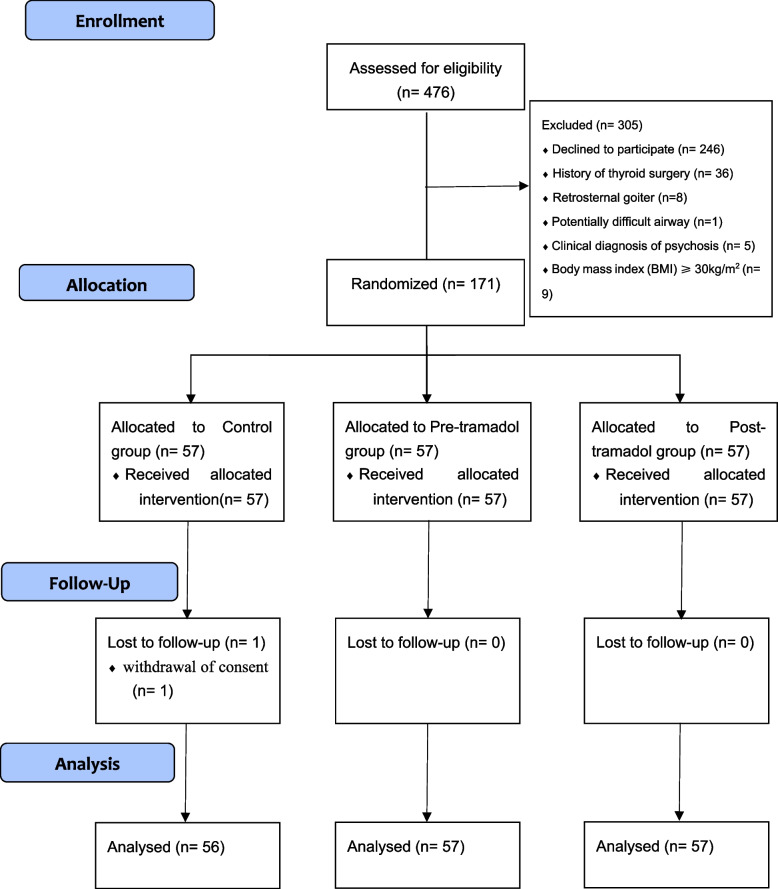
The CONSORT fow diagram for this study

Between September 10, 2021 and November 1, 2023, a total of 476 patients were screened for eligibility in the trial. Out of these, 171 patients were enrolled and randomly assigned to the study groups, as depicted in the flow diagram (Fig. 1). Post-experiment initiation, a single patient withdrew from the study, refusing to participate in follow-up assessments at both the 4 h and 24 h following extubation. Ultimately, the trial comprised 56 in Control group, 57 in Pre-tramadol group and 57 in Post-tramadol group.

Patient demographics and medical characteristics are detailed in Table 1. All participants achieved successful first-attempt intubation using video laryngoscopy.

**Table 1:** Demographic data and factors associated with postoperative sore throat

	Control (*n* = 56)	Pre-Tramadol (*n* = 57)	Post-Tramadol (*n* = 57)	*P*
Age, median (IQR), year	51.00 (42.50, 59.25)	48.00 (39.00, 55.00)	47.00 (39.00, 55.00)	0.164^b^
BMI, mean (SD), kg/m^2^	22.84 (3.17)	23.18 (3.18)	23.49 (2.95)	0.536^a^
ASA				0.078^c^
Ⅰ	26 (46.4)	28 (49.1)	17 (29.8)	
Ⅱ	30 (53.6)	29 (50.9)	40 (70.2)	
Cormack&Lehane grade				0.943^d^
Ⅰ	44 (78.6)	46 (80.7)	47 (82.5)	
Ⅱ	10 (17.9)	10 (17.5)	8 (14.0)	
Ⅲ	2 (3.6)	1 (1.8)	2 (3.5)	
Mallampati grade				0.750^d^
Ⅰ	41 (73.2)	47 (82.5)	42 (73.7)	
Ⅱ	12 (21.4)	8 (14.0)	11 (19.3)	
Ⅲ	3 (5.4)	2 (3.5)	4 (7.0)	
History of childbirth	55 (98.2)	53 (93.0)	52 (91.2)	0.349^d^
History of surgery	10 (17.9)	15 (26.3)	18 (31.6)	0.239^c^
Duration of operation, median (IQR), min)	46.50 (29.75, 60.25)	68.67 (60.87, 84.80)	65.17 (57.85, 76.00)	0.332^b^
The time needed for laryngoscopy, median (IQR), s	46.50 (29.75, 60.25)	37.00 (25.00, 56.00)	35.00 (20.00, 50.00)	0.132^b^
Intraoperative head movements	46 (82.1)	44 (77.2)	46 (80.7)	0.795^c^
Coughing or bucking in intraoperative period	5 (8.9)	6 (10.5)	4 (7.0)	0.846^d^
Coughing or bucking before extubation	18 (32.1)	17 (29.8)	18 (31.6)	0.962^d^
Coughing or bucking due to deflating cuff during extubation	27 (48.2)	30 (52.6)	26 (45.6)	0.750^d^
Coughing or bucking on the endotracheal tube	26 (46.4)	18 (31.6)	18 (31.6)	0.167^c^
Blood staining on the endotracheal tube after extubation	2 (3.6)	2 (3.5)	2 (3.5)	1.000^d^
Cough after extubation	3 (5.4)	1 (1.8)	5 (8.8)	0.249^d^
Trachea compressed by thyroid gland	8 (14.3)	11 (19.3)	11 (19.3)	0.723^c^
Left thyroidectomy				0.764^d^
None	14 (25.0)	14 (24.6)	20 (35.1)	
Partial	34 (60.7)	38 (66.7)	33 (57.9)	
Subtotal	4 (7.1)	3 (5.3)	2 (3.5)	
Total	4 (7.1)	2 (3.5)	2 (3.5)	
Right thyroidectomy				0.064^d^
None	16 (28.6)	28 (49.1)	14 (24.6)	
Partial	34 (60.7)	25 (43.9)	35 (61.4)	
Subtotal	1 (1.8)	2 (3.5)	1 (1.8)	
Total	5 (8.9)	2 (3.5)	7 (12.3)	
Thyroid isthmus resection				0.471^d^
None	2 (3.6)	1 (1.8)	4 (7.0)	
Partial	54 (96.4)	54 (94.7)	50 (87.7)	
Subtotal	0 (0.0)	1 (1.8)	1 (1.8)	
Total	0 (0.0)	1 (1.8)	2 (3.5)	
Lymph node dissection	30 (53.6)	22 (38.6)	34 (59.6)	0.069^c^
Intraoperative blood loss (IQR), ml	6.73 (3.00,5.00)	8.39 (3.00,5.00)	6.09 (3.00,6.00)	0.931^b^

Data are presented as No. (%) of patients unless otherwise indicated

^a^One-way analysis of variance

^b^Kruskal-Wallis test

^c^Chi-square test

^d^Fisher’s exact test

In our study cohort, the incidence of POST at rest was 31.18% in PACU after extubation, decreasing to 14.12% at 4 h after extubation and further to 5.29% at 24 h after extubation. In contrast, the incidence of POST on movement was higher, with 55.29% in PACU after extubation, peaking at 60.59% 4 h after extubation and then declining to 53.53% at 24 h after extubation.

### Primary outcome

Our primary outcomes reveal the incidence of POST at rest 24 h after extubation. As shown in Table 2, the rates were 7.1% Control group, 7.0% for Pre-tramadol group and lower at 1.8% for Post-tramadol group, although this difference was not statistically significant (*p* = 0.342). Similarly, the incidence of POST on movement at the same time point was 48.2% Control group, 63.2% for Pre-tramadol group and 49.1% for Post-tramadol group, with no significant difference observed (*p* = 0.201).

### Secondary outcomes

As shown in Table 2, the incidence of POST at rest in PACU after extubation was 23.2% for Control group, 35.1% for Pre-tramadol group and 35.1% for Post-tramadol group, with no statistically significant difference (*p* = 0.291). The incidence of POST on movement in PACU after extubation was 55.4% Control group, 61.4% for Pre-tramadol group and 49.1% for Post-tramadol group, again showing no significant difference (*p* = 0.419).

**Table 2:** The incidence and intensity of POST

	Control (*n* = 56)	Pre-Tramadol (*n* = 57)	Post-Tramadol (*n* = 57)	*P*
The incidence of POST at rest				
At PACU after extubation	13 (23.2)	20 (35.1)	20 (35.1)	0.291^c^
At 4 h after extubation	9 (16.1)	6 (10.5)	9 (15.8)	0.633^c^
At 24 h after extubation	4 (7.1)	1 (1.8)	4 (7.0)	0.364^d^
The incidence of POST on movement				
At PACU after extubation	31 (55.4)	28 (49.1)	35 (61.4)	0.419^c^
At 4 h after extubation	31 (55.4)	33 (57.9)	39 (68.4)	0.320^c^
At 24 h after extubation	27 (48.2)	28 (49.1)	36 (63.2)	0.201^c^
The intensity of POST at rest				
At PACU after extubation				0.630^d^
0	43 (76.8)	37 (64.9)	37 (64.9)	
1	8 (14.3)	11 (19.3)	9 (15.8)	
2	2 (3.6)	4 (7.0)	7 (12.3)	
3	3 (5.4)	5 (8.8)	4 (7.0)	
At 4 h after extubation				0.925^d^
0	47 (83.9)	48 (84.2)	51 (89.5)	
1	5 (8.9)	4 (7.0)	4 (7.0)	
2	3 (5.4)	3 (5.3)	1 (1.8)	
3	1 (1.8)	2 (3.5)	1 (1.8)	
At 24 h after extubation				0.167^d^
0	52 (92.9)	53 (93.0)	56 (98.2)	
1	4 (7.1)	3 (5.3)	0 (0.0)	
2	0 (0.0)	1 (1.8)	1 (1.8)	
3	0 (0.0)	0 (0.0)	0 (0.0)	
The intensity of POST on movement				
At PACU after extubation				0.915^c^
0	25 (44.6)	22 (38.6)	29 (50.9)	
1	15 (26.8)	15 (26.3)	13 (22.8)	
2	9 (16.1)	11 (19.3)	9 (15.8)	
3	7 (12.5)	9 (15.8)	6 (10.5)	
At 4 h after extubation				0.718^c^
0	25 (44.6)	18 (31.6)	24 (42.1)	
1	11 (19.6)	14 (24.6)	11 (19.3)	
2	10 (17.9)	15 (26.3)	15 (26.3)	
3	10 (17.9)	10 (17.5)	7 (12.3)	
At 24 h after extubation				0.667^d^
0	29 (51.8)	21 (36.8)	29 (50.9)	
1	12 (21.4)	18 (31.6)	15 (26.3)	
2	10 (17.9)	13 (22.8)	8 (14.0)	
3	5 (8.9)	5 (8.8)	5 (8.8)	

Data are presented as No. (%) of patients unless otherwise indicated

^a^One-way analysis of variance

^b^Kruskal-Wallis test

_c_Chi-square test

^d^Fisher’s exact test

Four hours after extubation, the incidence of POST at rest was 16.1% for Control group, 15.8% for Pre-tramadol group and 10.5% for Post-tramadol group, with no significant statistical difference observed (*p* = 0.633). The incidence of POST on movement at this time point was 55.4% for Control group, 68.4% for Pre-tramadol group and 57.9% for Post-tramadol group, with no significant difference (*p* = 0.320)(Table 2).

Regarding the intensity of POST, there was no statistically significant between the three groups at various time points, in PACU after extubation (at rest (*p* = 0.630) and on movement (*p* = 0.915)), at 4 h after extubation (at rest (*p* = 0.925) and on movement (*p* = 0.718)) and at 24 h after extubation (at rest (*p* = 0.167) and on movement (*p* = 0.673)) (Table 2).

For the intensity of discomfort of throat, there was no statistically significant difference between three groups in PACU after extubation (at rest (*p* = 0.579) and on movement (*p* = 0.903)), at 4 h after extubation (at rest (*p* = 0.871) and on movement (*p* = 0.786)) and at 24 h after extubation (at rest (*p* = 1.000) and on movement (*p* = 0.376)) (Table 3).

**Table 3:** The intensity of discomfort of throat

	Control (*n* = 56)	Pre-Tramadol (*n* = 57)	Post-Tramadol (*n* = 57)	*P*
The intensity of discomfort of throat at rest				
At PACU after extubation				0.579^d^
0	38 (67.9)	39 (68.4)	34 (59.6)	
1	13 (23.2)	10 (17.5)	12 (21.1)	
2	2 (3.6)	4 (7.0)	8 (14.0)	
3	3 (5.4)	4 (7.0)	3 (5.3)	
At 4 h after extubation				0.871^d^
0	43 (76.8)	44 (77.2)	46 (80.7)	
1	10 (17.9)	9 (15.8)	9 (15.8)	
2	3 (5.4)	2 (3.5)	1 (1.8)	
3	0 (0.0)	2 (3.5)	1 (1.8)	
At 24 h after extubation				1.000^d^
0	52 (92.9)	51 (89.5)	52 (91.2)	
1	4 (7.1)	5 (8.8)	4 (7.0)	
2	0 (0.0)	1 (1.8)	1 (1.8)	
3	0 (0.0)	0 (0.0)	0 (0.0)	
The intensity of discomfort of throat on movement				
At PACU after extubation				0.903^c^
0	19 (33.9)	20 (35.1)	23 (40.4)	
1	20 (35.7)	16 (28.1)	18 (31.6)	
2	10 (17.9)	12 (21.1)	11 (19.3)	
3	7 (12.5)	9 (15.8)	5 (8.8)	
At 4 h after extubation				0.786^c^
0	21 (37.5)	15 (26.3)	19 (33.3)	
1	14 (25.0)	17 (29.8)	16 (28.1)	
2	11 (19.6)	17 (29.8)	15 (26.3)	
3	10 (17.9)	8 (14.0)	7 (12.3)	
At 24 h after extubation				0.376^d^
0	19 (33.9)	17 (29.8)	23 (40.4)	
1	21 (37.5)	23 (40.4)	24 (42.1)	
2	10 (17.9)	14 (24.6)	5 (8.8)	
3	6 (10.7)	3 (5.3)	5 (8.8)	

Data are presented as No. (%) of patients unless otherwise indicated

^a^One-way analysis of variance

^b^Kruskal-Wallis test

^c^Chi-square test

^d^Fisher’s exact test

As shown in Table 4, the postoperative incision pain, as measured by VAS, revealed that the average VAS scores for incision pain at each time point were below 1.5 across all three groups. Notably, the differences in VAS scores between the groups were not statistically significant at the following time points: in PACU after extubation (*p* = 0.215), 4 h after extubation (*p* = 0.612), and 24 h after extubation (*p* = 0.707).

**Table 4:** The VAS of incision

	Control (*n* = 56)	Pre-Tramadol (*n* = 57)	Post-Tramadol (*n* = 57)	*P*
At PACU after extubation, median (IQR)	0.00 (0.00, 1.70)	1.00 (0.00, 1.50)	1.00 (0.00, 2.00)	0.215^b^
At 4 h after extubation, median (IQR)	0.00 (0.00, 1.62)	0.00 (0.00, 2.00)	0.00 (0.00, 2.00)	0.612^b^
At 24 h after extubation, median (IQR)	0.00 (0.00, 1.00)	0.00 (0.00, 1.00)	0.00 (0.00, 1.00)	0.707^b^

Data are presented as No. (%) of patients unless otherwise indicated

^a^One-way analysis of variance

^b^Kruskal-Wallis test

^c^Chi-square test

^d^Fisher’s exact test

As shown in Table 5, there was no statistically significant difference in the assessment of hoarseness in PACU after extubation (*p* = 0.745), at 4 h after extubation (*p* = 0.799) and at 24 h after extubation (*p* = 0.697). Two patients experienced severe hoarseness (aphasia) during postoperative PACU assessment, occurring in the Pre-tramadol group and Post-tramadol group, respectively. However, no severe hoarseness (aphasia) was observed at the follow-up of 24 h after extubation for these two patients.

**Table 5:** The severity of hoarseness, the incidence of vomiting and additional analgesics requirement

	Control (*n* = 56)	Pre-Tramadol (*n* = 57)	Post-Tramadol (*n* = 57)	*P*
The severity of hoarseness				
At PACU after extubation				0.745^d^
0	53 (94.6)	55 (96.5)	52 (91.2)	
1	0 (0.0)	0 (0.0)	1 (1.8)	
2	3 (5.4)	1 (1.8)	3 (5.3)	
3	0 (0.0)	1 (1.8)	1 (1.8)	
At 4 h after extubation				0.799^d^
0	50 (89.3)	54 (94.7)	54 (94.7)	
1	2 (3.6)	1 (1.8)	1 (1.8)	
2	4 (7.1)	2 (3.5)	2 (3.5)	
3	0 (0.0)	0 (0.0)	0 (0.0)	
At 24 h after extubation				0.697^d^
0	54 (98.2)	54 (94.7)	55 (96.5)	
1	0 (0.0)	2 (3.5)	0 (0.0)	
2	1 (1.8)	1 (1.8)	2 (3.5)	
3	0 (0.0)	0 (0.0)	0 (0.0)	
The incidence of additional analgesics requirement within 24 h after extubation	4 (7.1)	3 (5.3)	0 (0.0)	0.101^d^
The incidence of vomiting within 24 h after extubation	14 (25.0)	17 (29.8)	18 (31.6)	0.727^c^

Data are presented as No. (%) of patients unless otherwise indicated

^a^One-way analysis of variance

^b^Kruskal-Wallis test

^c^Chi-square test

^d^Fisher’s exact test

The incidence of additional analgesics requirement within 24 h after extubation in Pre-tramadol group (5.3%), Post-tramadol group (0%) was lower than that in Control group (7.1%) (*p* = 0.101), especially in Post-tramadol group (Table 5).

A common complication during the administration of tramadol is vomiting. As shown in Table 5, the incidence of vomiting within 24 h after extubation were recorded in Pre-tramadol group (29.8%), Post-tramadol group (31.6%) and Control group (25.0%) (*p* = 0.727) (Table 5).

## Discussion

In this randomized clinical trial comparing preoperative tramadol, intraoperative tramadol and placebo demonstrated comparable postoperative sore throat incidence across all study groups. Neither tramadol administration timing (pre- versus intraoperative) nor active treatment versus placebo showed meaningful differences in POST occurrence.

Numerous studies have identified female sex [15], larger endotracheal tube (ETT) size [14], high-volume/low-pressure cuff design [16, 17], excessive intracuff pressure [18], succinylcholine use and prolonged laryngoscopy [19] as independent risk factors for POST. In our study, baseline demographic characteristics and known POST-related risk factors were well-balanced across the three groups. Beyond conventionally reported factors, we prospectively recorded lesser-studied but plausible contributors, including intraoperative head movements and tracheal compression by the thyroid gland, both of which may intensify mechanical tracheal irritation. Furthermore, we systematically documented surgical history and pregnancy/fertility status, given their potential influence on postoperative pain perception and mucosal sensitivity.

Our sample size calculation referenced published the incidence of POST data (58%) following thyroidectomy with 7.0-mm ETT [3]. However, aiming to refine clinical relevance while maintaining statistical power, we deliberately selected a smaller 6.5-mm ETT, hypothesizing this intermediate size might mitigate POST severity without compromising airway safety. This choice proved judicious: our control group exhibited a 24-h the incidence of POST of 7.1% at rest, while swallowing-associated POST reached 48.2%, a rate consistent with expectations—higher than 6.0-mm ETT benchmarks (37%) yet markedly lower than 7.0-mm tube reports (58%).

To enhance study homogeneity and minimize confounding factors, we implemented strict standardization measures throughout the trial protocol. Key measures included: (1) exclusion of patients with anticipated difficult airways or complicated intubation; and (2) rigorous adherence to a standardized anesthesia regimen for all participants. Notably, after controlling for potential covariates associated with POST development, our observed the incidence of POST were substantially lower than those reported in previous studies [3, 6], especially at rest.

Multiple pathogenic mechanisms have been implicated in postoperative sore throat (POST) development, primarily including (1) airway inflammatory responses, (2) mechanical trauma from endotracheal intubation and (3) cuff pressure-induced mucosal ischemia [20]. In clinical practice, diverse pharmacological and non-pharmacological preventive strategies have been consequently developed to mitigate POST occurrence. [3, 8].

Strategies for preventing and reducing POST encompass two distinct approaches: pharmacological agents targeting airway inflammation and non-pharmacological interventions designed to minimize mechanical trauma to the upper airway mucosa [8]. commonly used pharmacological options include NSAIDs [21], lidocaine [9], dexmedetomidine [22] and dexamethasone [2]. Notably, the efficacy of selective anti-inflammatory drugs—even those lacking analgesic properties—in reducing the incidence of POST further reinforces the central role of inflammatory pathways in POST pathogenesis.

While previous studies have demonstrated that topical tramadol application to oral and pharyngeal mucosa significantly reduces the incidence of POST [10–12]. our findings indicate that intravenous tramadol administration does not confer comparable protective effects. We hypothesize that the underlying mechanism likely involves that the pathophysiology of POST involves both inflammatory mediators and mechanical trauma mechanisms. While tramadol has systemic analgesic properties, its limited anti-inflammatory activity may explain its ineffectiveness when administered intravenously. In contrast, topical tramadol demonstrated benefit in previous studies, suggesting a local anesthetic and anti-inflammatory action are necessary to mitigate POST. Similar results appear in intravenous diclofenac had no effect in patients undergoing laparoscopic surgery [23], but topical diclofenac reduced the incidence of POST following caesarean section [24]. Ketamine, NMDA receptor antagonists, is likely due to a local effect, because an intravenous infusion had no effect on the incidence or severity of POST following cholecystectomy [25].

These findings imply that administration route (topical vs. intravenous) critically determines the therapeutic efficacy of tramadol, ketamine and diclofenac against POST, with topical delivery demonstrating superior effectiveness. This underscores the necessity of systematically evaluating administration routes when investigating pharmacological interventions for POST prevention.

VAS of postoperative incision pain remained low (< 1.5) across groups suggesting that uniform administration of fentanyl (1 μg/kg) at the initiation of surgical wound irrigation ensured comparable baseline analgesia across groups, which may have masked any incremental systemic analgesic effect of tramadol. However, because POST is more strongly associated with local mucosal inflammation than with surgical pain, the lack of effect is likely attributable to tramadol’s limited anti-inflammatory activity rather than fentanyl confounding alone. Notably, tramadol was associated with numerically more vomiting, though not significant.

Although tramadol has been associated with an increased bleeding risk, existing evidence originates from scenarios involving prolonged administration [26], concomitant oral anticoagulants [27] or concurrent vitamin K therapy [28]. In the present study, none of these confounders were present: enrolled patients had no history of long-term tramadol use oral anticoagulation or vitamin K supplementation. Furthermore, our protocol employed only a single intra-operative dose of tramadol and no inter-group differences in intraoperative blood loss were observed. No patient required unscheduled reoperation for bleeding during the postoperative period.

Our findings suggest that prophylactic intravenous tramadol administration does not significantly reduce the incidence of POST in thyroid surgery. Therefore, its routine use for POST prevention appears unjustified in this context. However, tramadol remains clinically valuable for postoperative pain management, where its administration should follow established analgesic guidelines.

This study has several limitations that should be acknowledged: First, although we hypothesize that intravenous tramadol's inability to reduce the incidence of POST may stem from its lack of significant anti-inflammatory effects, we did not measure systemic (e.g. serum pro-inflammatory cytokines) or local (e.g., tracheal mucosal cytokine levels) inflammatory markers to validate this potential mechanism. Second, our study only examined a single intravenous tramadol dosage. The potential dose-dependent effects of IV tramadol—as well as its topical administration—on the incidence of POST remain unexplored under comparable experimental conditions. Third, while the Post-tramadol group demonstrated a numerically lower the incidence of POST compared to the Control and Pre-tramadol groups (Table 2), this trend did not achieve statistical significance (*p* = 0.342). However, the observed absolute differences suggest a possible biological effect that current sample size limitations may have obscured. Given the marginal p-value, future studies with larger cohorts are warranted to enhance statistical power and more reliably determine whether tramadol administration timing truly influences the incidence of POST.

## Conclusion

Our findings demonstrate that intravenous tramadol (1 mg/kg) does not significantly reduce the incidence of POST following thyroid surgery. Future investigations should explore: (1) alternative tramadol dosing regimens, (2) different administration routes (particularly topical application) and (3) conduct larger-scale trials to enhance statistical power and detect potential treatment effects that may have been obscured by sample size limitations in the current study.

## Data Availability

The datasets generated during and/or analyzed during the current study are available from the corresponding author (Xiao-Dan Wu) upon reasonable request.

## Abbreviations

POST: Postoperative sore throat
PACU: Post anesthesia care unit
ASA: American Society of Anesthesiologists
NMDA: N-Methyl-D-Aspartic Acid
VAS: Visual Analogue Scale
ETT: Endotracheal tube
